# Bone Marrow Is a Reservoir for Cardiac Resident Stem Cells

**DOI:** 10.1038/srep28739

**Published:** 2016-06-27

**Authors:** Na Liu, Xin Qi, Zhibo Han, Lu Liang, Deling Kong, Zhongchao Han, Shihua Zhao, Zuo-Xiang He, Zongjin Li

**Affiliations:** 1 Nankai University School of Medicine, Tianjin, China; 2 The Key Laboratory of Bioactive Materials, Ministry of Education, Nankai University, the College of Life Science, Tianjin, China; 3 Department of Cardiology, Tianjin Union Medical Center, Nankai University Affiliated Hospital, Tianjin, China; 4 State Key Lab of Experimental Hematology, Institute of Hematology & Hospital of Blood Diseases, Chinese Academy of Medical Sciences, Tianjin, China; 5 Beijing Institute of Health and Stem Cells, No. 1 Kangding Road, BDA, Beijing, China; 6 Department of Radiology, Fu Wai Hospital, Chinese Academy of Medical Sciences and Peking Union Medical College, Beijing, China; 7 Department of Nuclear Medicine, State Key Laboratory of Cardiovascular Disease, Fu Wai Hospital, National Center for Cardiovascular Disease, Peking Union Medical College & Chinese Academy of Medical Sciences, Beijing, China

## Abstract

Resident cardiac stem cells (CSCs) represent a responsive stem cell reservoir within the adult myocardium and have a significant function in myocardial homeostasis and injury. However, the distribution, origin, homing and possible therapeutic benefits of CSCs are still under discussion. Here we investigated whether bone marrow (BM) stem cells could contribute to repopulating the pool of CSCs in heart. The engraftment of BM cells in heart was detected at a low level after BM transplantation (BMT) and ischemia/reperfusion (I/R) could increase BM cells engraftment but not significant. We clarified that more than 50% CSCs are derived from BM and confirmed that BM-derived CSCs have similar characteristics with the host CSCs. Furthermore, we transplanted BM-derived CSCs into heart ischemia models and presented evidence for the first time that BM-derived CSCs can differentiate into cardiomyocytes *in vivo*. In conclusions, BM stem cells could be a potential back-up source of CSCs for restoring heart function after injury or maintaining homeostasis of CSCs.

The mammalian heart is believed to grow by enlargement but not proliferation of cardiomyocytes (CMs) during post-natal development. However, the long-held belief of the heart as post mitotic organ has been toppled by demonstration of myocyte turnover throughout life and the existence of adult cardiac stem cells (CSCs)^1^,^2^. The majority of studies indicate that CMs renewal rate is very low, less than 1% per year, and the rate declines with age^3^. The mechanism for cardiomyocyte homeostasis in normal mammalian myocardium is potentially different from regeneration after injury, which could trigger a cascade of signals that result in activation of dormant progenitor cells or proliferation of existing cardiomyocytes^3^. Previous works have demonstrated that CSCs can contribute to new CMs and vascular lineages after injury^4^ and also improve cardiac function after CSCs injected into the infarct zone^5^.

Adult CSCs in the myocardium have been identified using a variety of approaches, including the expression of surface markers such as c-Kit, Sca-1, and physiological properties such as the ability to efflux fluorescent dye or to form multicellular spheroids (cardiosphere)^3^,^5^,^6^,^7^. When transplanted into injured myocardium, CSCs can differentiate into functional cardiomyocytes and all the major cardiac cell lineages^8^. This rare population cells reside in specific anatomic regions of the myocardium, the so-called CSC niches, are considered both to be at the helm of the cardiac turnover and to play a fundamental role in the adaptive response of the myocardium to cardiac injury^9^. An increasing body of literature has shown that CSCs can attenuate left ventricular remodeling and improve heart function in animal myocardial infarction models^5^,^10^. Moreover, the Phase I clinical trials of the application of these cells in clinical settings has been carried out recently^5^.

Studies have revealed that bone marrow (BM)-derived cells can improve heart function in model of myocardium ischemia^11^ and hematopoietic stem cells can transdifferentiate into CMs in mouse BM transplantation (BMT) model^12^,^13^,^14^. Moreover, several studies have shown a constant exchange of hematopoietic stem cells between BM and peripheral blood and it has been estimated that up to 400 hematopoietic stem cells circulate in the blood of a mouse at any one time^15^. Therefore, we are curious whether BM cells contribute to repopulating the pool of CSCs in heart. In this study, we examined cardiomyogenic potential of BM cells following syngeneic BM transplantation. Moreover, the cardiomyogenic potential of BM cells was investigated and the characteristics of BM-derived CSCs were clarified. In this study, the phase-bright cells were termed as CSCs as reported previously^6^,^14^,^16^,^17^. Furthermore, we transplanted BM-derived CSCs into mouse heart ischemia model to investigate their therapeutic potential *in vivo*.

## Results

### Engraftment of BM stem cells

**Figure 1:**
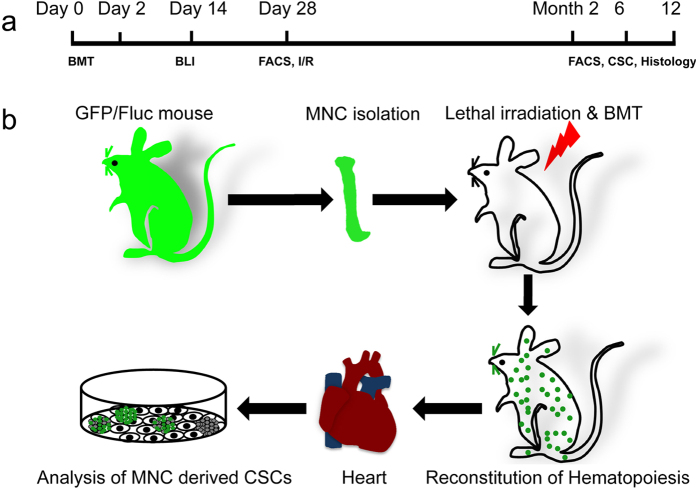
Schedule for experiments and schematic of mouse model. (**a**) Schedule of this study. Bone marrow transplantation (BMT) was carried out at day 0, and bioluminescence imaging (BLI)/FACS was used for tracking hematopoietic reconstruction. To investigate if injury can increase transdifferentiation of BM stem cells into cardiac phenotype, I/R (ischemia reperfusion) surgery was did at day 28. The hearts of BMT mice were subjected to enzymatic digestion for culture CSCs at month 2, 6, and 12. The engraftment and transdifferentiation of BM cells in myocardium were analyzed by FACS and immunohistology at month 2, 6, and 12 after BMT. (**b**) An outline of the protocol used for the isolation of BM derived CSCs. Wild type mice were lethal irradiated and transplanted BM mononuclear cells from GFP/Fluc mice. The hearts were subjected to enzymatic digestion, cultured after hematopoietic reconstruction. GFP positive phase-bright cells appeared over adhered fibroblast-like cells were identified as BM-derived CSCs.

**Figure 2:**
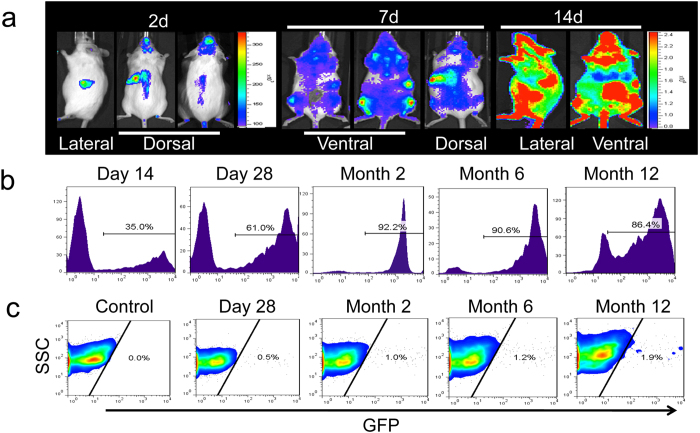
Engraftment of BM stem cells. (**a**) Dynamics of BM stem cells engraftment from Fluc/GFP FVB mice. (**b**) Longitudinally analysis of GFP positive cells in peripheral blood after BMT at multiple time points by FACS. All experiments were performed in triplicate each time point. (**c**) Longitudinally analysis of GFP positive cells in heart after BMT by FACS. All experiments were performed in triplicate at each time point (n = 3).

To study the pattern and dynamics of BM engraftment, whole BM cells were transplanted into lethally irradiated nontransgenic, syngeneic recipients (Fig. 1). Bioluminescent foci were detected as early as 2 days after transplantation and the most frequently observed locations were at anatomic sites corresponding to the locations of the spleen, skull, vertebrae, femurs, and sternum, as well as from other BM compartments. Bioluminescence imaging (BLI) revealed a significant degree of hematopoietic reconstitution at day 14 (Fig. 2a) and remained high thereafter (data not shown). Furthermore, hematopoietic reconstruction of recipient mice were confirmed by robust expression of GFP from 35% at day 14 to more than 92% at month 2 and kept around 90% in peripheral blood by FACS analysis (Fig. 2b). However, the engraftment of GFP+ cells into heart was at low level, around 1–2% from month 2 to month 12 and I/R could increase GFP+ cells engraftment but not significant (Figs 2c and S1).

### BM-derived CSCs

**Figure 3:**
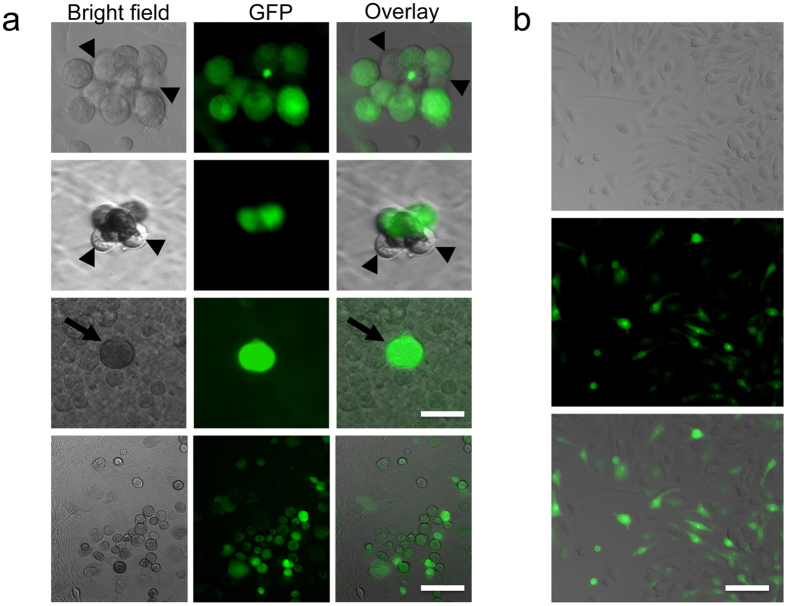
Isolation and culturing of CSCs after BMT. (**a**) After a period ranging from 1 to 3 weeks, phase-bright cells migrated over a layer of fibroblast-like cells. The clump of phase-bright cell includes GFP positive cells (arrow) and GFP negative cells (arrow head). GFP expression indicates the cells are BM originated. Up three panel, scale bar = 10 μm; lower panel, scale bar = 50 μm (**b**) Subculture of phase-bright cells. The results revealed that this population cells are mixture of GFP positive and negative cells. Morphology of CSCs cultured in poly-D-lysine-coated plates (up), expressing GFP (middle), and overlay (bottom). Scale bar = 50 μm. Totally 10 hearts were harvested for CSCs culture at month 6.

**Figure 4:**
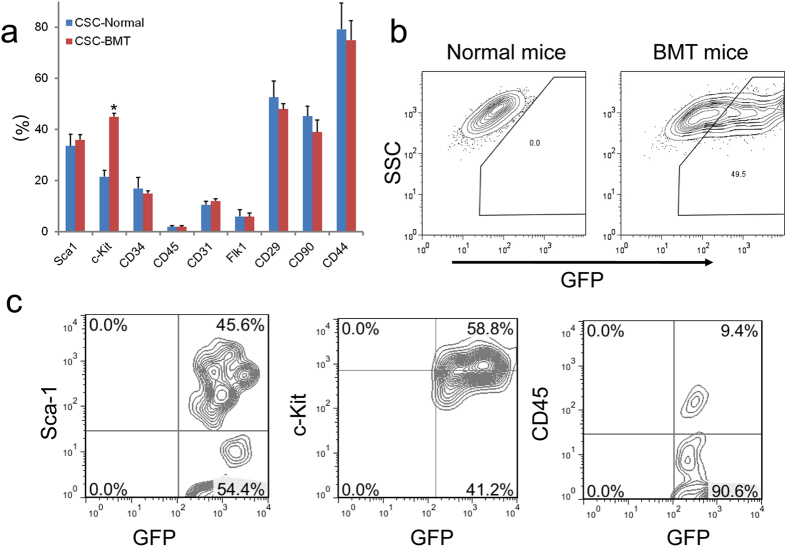
Characterization of surface markers in CSCs. (**a**) Quantitative analysis by FACS of CSC phase-bright derived from BMT mice hearts. CSCs from normal mice were used as control. CSCs from BMT mice heart have same expression pattern compared to CSCs form normal mice, but higher level of c-Kit. (**b**) Phase-bright CSCs from BMT mice hearts are about 50% GFP positive. (**c**) GFP positive phase-bright CSCs express same levels Sca-1, c-Kit and CD45 compared with mixed population CSCs from BMT mice hearts. All experiments were performed in triplicate (n = 3).

The hearts were subjected to enzymatic digestion, cultured after BMT. Phase bright spherical cells that spontaneously separated from the myocardium samples were identified after 2–3 weeks. These cells demonstrated a high GFP expression ratio (Fig. 3a). This result indicates that BM-derived stem cells can differentiate into CSCs and acquire cardiac fate within the host heart. The morphology of subcultured bright cells (Fig. 3b) was consistent with previous report^16^,^17^,^18^,^19^. In order to further characterize their cellular phenotypes, detailed flow cytometry analysis was performed. Overall, compared with CSCs isolated from wild type mice, CSCs from BMT mice have a similar expression pattern, but express higher c-Kit, suggesting similar cellular characteristics of CSCs from BM (Fig. 4a). Quantitative analysis by FACS revealed that around half bright cells is GFP positive, which indicates this population cells are from BM (Fig. 4b). Further analysis showed that GFP+ CSCs from BMT mice have a similar expression pattern of CSCs markers Sca-1, c-Kit and hematopoietic lineage marker CD45 compared to whole bright cell population (CSCs), but a little higher Sca-1 and CD45 (Fig. 4c). Moreover, the expression of these markers in GFP+ CSCs from BM is similar to previously published data^6^,^18^,^19^. Furthermore, engraftment of BM cells in heart after BMT and BM-derived CSCs were enhanced with the time prolonging (Figs S2 and S3).

### Characterization and Differentiation of BM-derived CSCs

**Figure 5:**
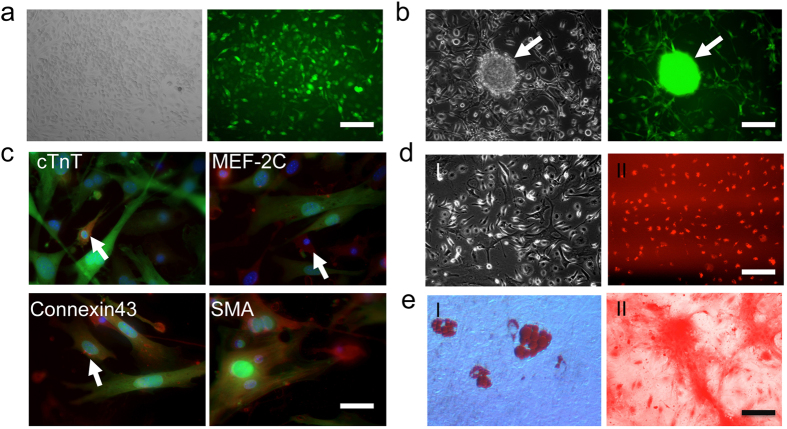
Multipotent capacity of CSCs. (**a**) Subculture the sorted GFP positive CSCs. Morphology of CSCs cultured in poly-D-lysine-coated plates (left) and expressing green fluorescence protein (right). (**b**) With cardiac differentiation medium, some CSCs can form cardiac sphere (arrow) with GFP expression. (**c**) Cardiac and smooth muscle differentiation of CSCs *in vitro*. Immunostaining of GFP positive CSCs with cardiac troponin T (cTnT), myocyte enhancer factor 2C (MEF-2C), connexin-43, and α-smooth muscle actin (α-SMA). (**d**) Endothelial differentiation of CSCs *in vitro*. The cells were cultured in EGM-2 medium with 10 ng/ml VEGF and showed endothelial differentiation by morphology (**I**) and uptake of Dil-ac-LDL (**II**). (**e**) Oil red staining shows adipogenic differentiation of the CSCs induced for 2 weeks. (**I**) Alizarin red S staining of calcium shows CSCs induced to differentiate into osteoblasts (**II**). (**a**,**b**,**d**,**e**) scale bar = 20 μm; (**c**), scale bar = 10 μm. All experiments were performed in triplicate (n = 3).

The GFP+ CSCs were sorted by FACS for further experiment. These population cells are GFP positive and further can form cardiac sphere in cardiac differentiation medium (Fig. 5a,b). We next examined the differentiation of BM derived CSCs into various cell types. Similar to other studies^6^,^20^, these cells can differentiate into cardiomyocytes and smooth muscle cells, as documented by positive staining for cardiac troponin T (cTnT), connexin 43, myocyte enhancer factor-2C (MEF-2C), and α-smooth muscle actin (Fig. 5c). In addition, these cells were capable of differentiating into endothelial cells as demonstrated by DiI-ac-LDL uptake (Fig. 5d), into adipocytes as demonstrated by oil red staining (Fig. 5e,I), and into osteoblasts as demonstrated by Alizarin Red S staining (Fig. 5e,II).

### BM-derived cells can acquire cardiac properties

**Figure 6:**
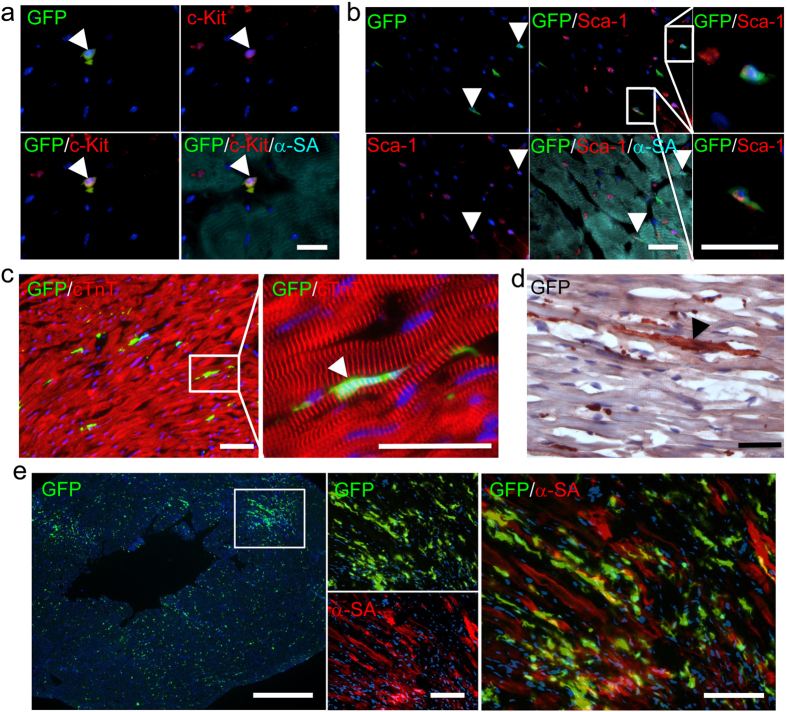
The cardiac fate of BM-derived stem cells. BM-derived cells contributed to the c-Kit+ (**a**) or Sca-1+ (**b**) CSCs confirmed by triple staining with GFP and α-sarcomeric actin (α-SA). (**a**,**b**) Scale bar = 10 μm. Totally 10 hearts have been harvested for staining at month 6. (**c**) BM-derived stem cells can differentiate and acquire of cardiac fate after the engraftment within the host heart as confirmed by cTnT and GFP double stainings (n = 5). Scale bar = 20 μm. (**d**) BM-derived stem cells can differentiate into cardiomyocytes and were integrated structurally with resident myocytes as confirmed by GFP staining (n = 5). Scale bar = 20 μm. (**e**) The cardiac fate of BM-derived stem cells was enhanced by I/R injury as confirmed by α-SA and GFP double stainings. Left, scale bar = 200 μm; Middle and right, scale bar = 20 μm.Totally 10 hearts were harvested for staining at month 6 after I/R.

Maintenance and trafficking of stem cells is critical for organogenesis during development and for homeostasis and regeneration in adulthood^21^,^22^. CSCs have been identified in the adult heart, and the myocardium possesses interstitial structures with the architectural organization of stem cell niches that harbor CSCs^23^. To investigate if the continuous trafficking hematopoietic stem cells in blood stream can fill CSCs niches and contribute to the maintenance of normal heart function and restoring degraded myocardium, we performed immunostaining to determine phenotypic plasticity of BM derived cells. Our results revealed that BM-derived stem cells can differentiate and acquire a cardiac fate after the engraftment within the host heart. Six months after BMT, immunostaining demonstrated that BM-derived cells contributed to the Sca-1+ or c-Kit+ CSCs, although most CSCs came from indigenous cells (Fig. 6a,b). Moreover, most BM-derived stem cells were CD45-negative (data not shown). Furthermore, transplanted BM-derived stem cells can differentiate into cardiomyocytes and integrate structurally with resident myocytes (Fig. 6c,d). The cardiac fate of BM-derived stem cells was enhanced by I/R injury or as time extends, which suggesting that the cardiac microenvironment may change the fate of BM-derived stem cells, and BM cells will lose their hematopoietic phenotype and acquire cardiac lineage (Fig. 6e). However, no overt increase of Sca-1+ and c-Kit+ CSCs was detected after I/R injury (data not show), which indicates the rate of cardiac transdifferentiation of BM-derived stem cells is low. Furthermore, endothelial, neural, skeletal muscle, intestinal, and renal linages transdifferentiation of BM-derived cells were also observed (Figs S4 and S5).

### *In vivo* characterization of BM-derived CSC

**Figure 7:**
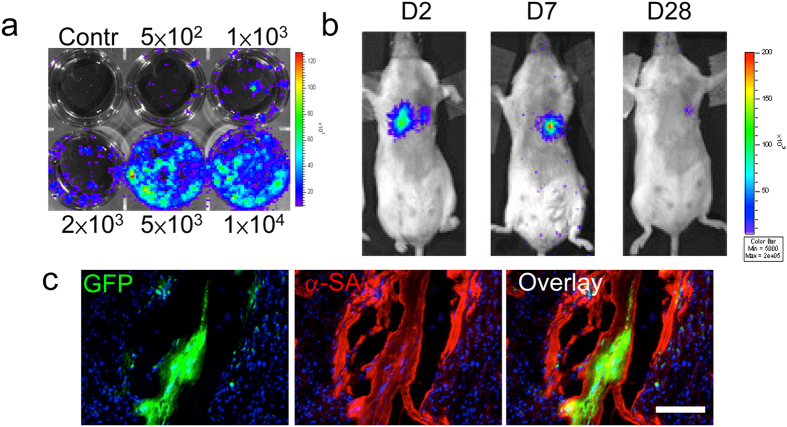
Transplantation of BM-derived CSCs. (**a**) *Ex vivo* imaging analysis of CSCs shows increasing BLI signals with cell numbers (n = 3). (**b**) Reporter gene imaging of CSCs fate after transplantation (n = 15). A representative animal injected with 5 × 10^5^ CSCs shows significant bioluminescence activity at day 2, which decreases progressively over the following 4 weeks. (**c**) Tracking of grafted CSCs by immunofluorescence at week 2 (n = 7). Transplanted CSCs can differentiate and integrate with host myocardium as confirmed by GFP and α-sarcomeric actin (α-SA) double staining. Scale bar = 20 μm.

CSCs have been isolated and these cells offer a provocative method to regenerate the damaged myocardium^6^. *In vitro* assessment of firefly luciferase (Fluc) expression showed the correlation between the total cell numbers and the bioluminescence signals (Fig. 7a), which is needed for accurate tracking of cell fate in subsequent *in vivo* imaging studies. We were intrigued if transplantation of BM-derived CSCs can benefit cardiac function recovery after I/R injury. In order to address this question, adult wild type FVB mice were subjected to I/R injury followed by injection with 5 × 10^5^ cultured GFP+ CSCs from BMT FVB mice. Injection of CSCs into infarcted myocardium resulted in a robust BLI signal at day 2. However, serial imaging of the same animals out to 4 weeks demonstrated a significant decay in the BLI signal (Fig. 7b) and it is consistent with previous report^6^. Histologic analysis of the myocardium was performed and the results revealed that GFP+ CSCs from BMT FVB mice could differentiate into cardiomyocytes as confirmed by α-SA and GFP double staining at day 14 (Fig. 7c). Taken together, the BM derived CSCs have similar characteristics and are resemble with previous reports about CSCs^6^,^16^,^24^,^25^.

## Discussion

**Figure 8:**
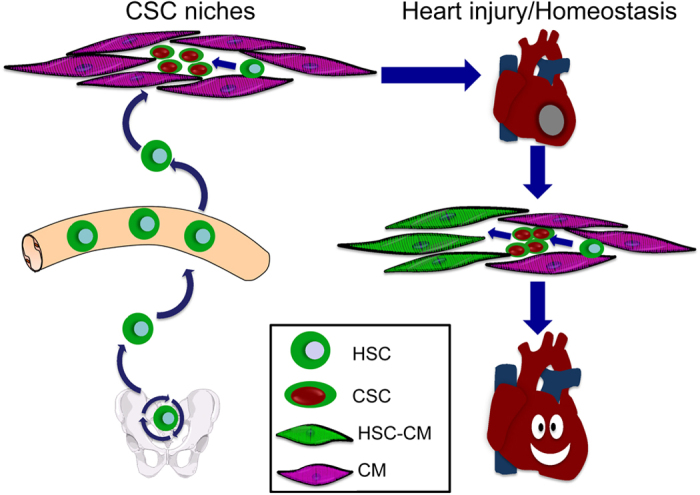
Trafficking of BM stem cells in circulation and heart. BM stem cells could be a potential back-up source of CSCs for restoring the function of ischemia heart disease or maintaining the homeostasis of CSCs.

In this study, we confirmed the cardiomyogenic potential of BM cells following syngeneic BM transplantation. Our data revealed that the engraftment of GFP+ cells into heart was at low level, around 1–2% from month 2 to month 12 and I/R could increase GFP+ cells engraftment but not significant. Moreover, we clarified that more than 50% CSCs are derived from BM with phase-bright culture methods and confirmed that BM-derived CSCs have similar characteristics with host CSCs. Furthermore, our result demonstrated here for first time that BM-derived CSCs can differentiate into cardiomyocytes *in vivo* in heart ischemia models. We conclude that BM stem cells could be a potential back-up source of CSCs for restoring heart function after injury or maintaining the homeostasis of CSCs (Fig. 8).

It has been estimated that cardiomyocytes renew range from less than 1% per year to more than 4% per year, either by the division of pre-existing cardiomyocytes or the differentiation of CSCs^26^,^27^. Previous works on CSCs have demonstrated significant improvement in cardiac function after CSCs injection, and a lot of evidences have shown that resident CSCs could turn into cardiomyocytes directly *in vivo*^28^. Moreover, two Phase I clinical trials have been performed recently^5^. Endogenous CSCs are characterized based on c-Kit, Sca-1, Isl-1, cardiospheres, side population and others^5^,^25^,^29^. CSCs represent a responsive stem cell reservoir within the adult myocardium and recent studies have revealed that CSCs have a significant function in myocardial homeostasis and after injury^5^,^27^,^30^,^31^. Thus, it was assumed that if the heart loses a number of CMs, CSCs would have to sustain the heart function at least in part. However, the distribution, origin, and possible therapeutic benefits of CSCs are still under discussion^25^. New investigations are needed to identify the distribution and localization of the resident stem/progenitor cell population in the heart, and most importantly to reveal their origin and homing in the heart.

Continuous trafficking of BM stem cells among the organs and circulation likely fill empty or damaged niches, contribute to the maintenance of normal organ function and restoring degraded tissues^22^,^32^,^33^. Moreover, differentiation of BM cells into cardiomyocytes (CMs) also gave rise to the thought that the BM-derived stem cells population is critical for myocardium homeostasis and repair after injury via reprogramming the phenotype of hematopoietic stem cells to induce CM renewal^33^. This strategy may ultimately win over cell transplantation because of the challenges of timely production of sufficient quantities of autologous cells that meet all criteria necessary for safe and efficacious transplantation^3^. In this study, the transplanted BM stem cells migrate via the bloodstream throughout all experimental courses for several months, which would increase the possibility of BM-derived stem cells residing in heart and our results revealed that the BM-derived hematopoietic stem cells can reside in myocardium and differentiate into mature myocytes.

Currently, several approaches have been used for CSCs isolation, which build a foundation for our understanding of the developmental logic of cardiac regeneration^5^. In this study, isolation of CSCs was based on phase bright cells growing as self-adherent clusters from sub-cultures of murine heart^6^,^16^. We found that CSCs from BMT mice have a similar expression pattern compared with cells isolated from wild type mice, suggesting BM stem cells contribute to CSCs pool. Further study should be done to extend this observation to other kind of CSCs with different isolation protocols. Considering engraftment of GFP+ BM cells into heart at 1–2% from month 6 to 12, this study indicates that BM stem cells do not have a significant function in myocardial homeostasis and suggests that their role after injury is also limited. Recent study also revealed that c-Kit-positive CSCs originating in the heart generated new cardiomyocytes at a percentage of 0.03 or less^34^. Our results revealed that BM-derived Sca-1+, c-Kit+ CSCs can be found resident in myocardium *in situ* and can be expanded *in vitro*. Considering low engraftment and cardiac differentiation of BM-derived stem cells, we must achieve a deeper understanding of the molecular mechanisms of BM stem cells trafficking and residency for further exploiting the potential of this therapeutics.

Previous studies have shown that BM cells can transdifferentiate into cardiomyocytes *in vivo* although this notion has been disputed^35^. Other than directly cardiac differentiation of BM cells to mend a broken heart by repairing damaged muscle, BM cells may help it in a more indirect way, transdifferentiating of BM cells into CSCs. Strategies to enhance the transdifferentiation of BM stem cells into CSCs and CMs may consolidate cell-based therapies for heart injury. Promoting BM stem cell mobilization is a common strategy to augment the cellular yield of peripheral blood apheresis for clinical stem cell transplantation, and a similar approach has been suggested to increase the number of circulating cells available for homing after injury^32^,^36^. Because the current study shows that BM stem cells at least have some limited ability to add into CSCs pool and regenerate contractile heart cells, it may be possible to find a method to enhance this ability so the circulating BM stem cells can eventually be used in a truly therapeutic application. A complex signaling network likely underlies the selective recruitment of the aforementioned BM stem cell populations following injury^33^. C-X-C chemokine receptor type 4 (CXCR-4), a G-protein-coupled seven-transmembrane receptor, together with its ligand SDF-1, can play a crucial role during homing of stem cells at the site of injury, and preservation of the injured tissue^25^. Together with our report of cardiac homeostasis and regeneration by BM stem cells, these studies provide a set of models for increasing therapeutic efficiency of trafficking BM stem cells.

In conclusion, our results revealed that BM serves as a reserve pool of CSCs based on phase-bright culture methods. Following hematological reconstitution and I/R, BM-derived cells can contribute to the Sca-1+ or c-Kit+ CSCs population and transdifferentiate into CMs, although the transition of phenotype is at low level. Moreover, BM-derived CSCs have the same characteristics with host CSCs and can increase myocardial function in heart ischemia models. We can conclude that BM stem cells could be a potential back-up source of CSCs for restoring heart function after injury or maintain the homeostasis of CSCs. However, strategies to enhance the transdifferentiation of BM stem cells into CSCs and CMs, will be the key to maximizing therapeutic effect and producing a clinically relevant therapy.

## Materials and Methods

A detailed description of the experimental procedures is provided in the Online Supplement.

### Animals

The FVB-GFP/Fluc mice were obtained by mating FVB-GFP mice with FVB-Fluc mice. The luciferase transgenic mouse line (FVB-Fluc), expressing firefly luciferase (Fluc) under the control of the widely expressed β-actin promoter, was kindly provided by Xenogen Corporation (Alameda, CA). The green fluorescent protein (GFP) transgenic mouse line (FVB-GFP), expressing GFP under the control of the widely expressed β-actin promoter, was kindly provided by Professor Baojin Wu^37^. Wild type FVB mice were purchased from the Laboratory Animal Center of the Academy of Military Medical Sciences (Beijing, China). Animal protocols were approved by the Nankai University Animal Care and Use Committee, which conform to the Guide for the Care and Use of Laboratory Animals published by the US National Institutes of Health (8^th^ edition, 2011).

### Isolation and transplantation of bone marrow cells

Eight to 10-week-old female FVB mice were irradiated with 12.0 Gy of γ-irradiation in 2 divided doses, 2 hours apart, on the day of surgery. Bone marrow (BM) cells were isolated from the femur and tibia of 8 to 10-week-old female transgenic FVB-GFP/Fluc mice, expressing the β-actin-GFP and Fluc genes. For bone marrow transplantation (BMT), wild-type irradiated mice were injected with 0.2 ml PBS with or without 2.0 × 10^5^ BM mononuclear cells via tail veins at 2 hours after irradiation.

### Bioluminescence imaging

Bioluminescence imaging (BLI) was performed on all animals using the Xenogen IVIS Lumina II system (Xenogen, Alameda, CA). After intraperitoneal injection of the reporter probe D-Luciferin (150 mg/kg), animals were imaged for 1–10 minutes. Bioluminescence signal was quantified in units of maximum photons per second per cm square per steradian (photons/sec/cm^2^/sr) as described^38^,^39^. Dorsal, ventral, and two lateral images were obtained from each animal at each time point to better determine the origin of photon emission.

### Ischemia/perfusion (I/R) surgery

To explore if ischemia can increase transdifferentiation efficiency of BM-derived cells, I/R surgery was done at day 28 after BMT (n = 15). The I/R heart model was performed by ligation of mid-left anterior descending (LAD) artery for 30 minutes, and then followed by reperfusion. Infarction was visually confirmed by blanching of the anterolateral region of the left ventricle along with dyskinesis^40^,^41^.

### Isolation and culture of CSCs

CSCs were isolated from BMT mice as described^6^,^16^,^17^,^18^,^19^. In brief, myocardial tissue was cut into 1- to 2-mm piece, washed with Hanks’ balanced salt solution (HBSS) (Invitrogen, Carlsbad, CA), and incubated with 0.1% collagenase II for 30 minutes at 37 °C with frequent shaking. Cells were then filtered through 100-μm mesh. The cardiac cell samples were either directly evaluated for the expression of GFP by FACS or subcultured as described^6^,^16^. After 2 to 3 weeks, a population of phase-bright cells appeared over the adhered fibroblast-like cells. These phase-bright cells were collected by two washes with PBS, and one wash with cell dissolution buffer (Gibco, Grand Island, NY) at room temperature under microscope monitoring, GFP+ cells were sorted by FACS, and sub-cultured in poly-lysine coated plates (BD Biosciences) with the same medium. An outline of the experiments is depicted in Fig. 1.

### Flow cytometry analysis

FACS analysis of the bright cells from BMT mice were carried out and the bright cells from normal FVB mice were used as control. Antibodies used in this study were phycoerythrin (PE) conjugated anti-CD34, CD29, CD90, CD44, Sca-1, CD45, Flk1, Allophycocyanin (APC) conjugated anti-CD31 and c-Kit (all from BD Pharmingen). The stained cells were analyzed using FACS LSR (Becton-Dickinson, MA). Dead cells stained by propidium-iodide (PI) were excluded from the analysis. Isotype-identical antibodies served as controls (BD Pharmingen). FlowJo software (Tree Star Inc, Ashland, OR) was used for followed data analysis.

### *In vitro* differentiation of CSCs

For cardiac and smooth muscle differentiation, CSCs were cultured in poly-D-lysine-coated plates in differentiation medium containing 35% Iscove modified Dulbecco medium with 10% fetal bovine serum/65% Dulbecco modified Eagle medium-Ham F-12 mix containing 2% B27, 0.1 mmol/L 2-mercaptoethanol, 10 ng/mL epidermal growth factor (R&D Systems, Minneapolis, MN), 20 ng/mL basic fibroblast growth factor, 40 nmol/L cardiotrophin-1, 40 nmol/L thrombin (Sigma-Aldrich, St Louis, MO), 100 U/mL penicillin G, 100 g/mL streptomycin, and 2 mmol/L glutamine. For endothelial differentiation, CSCs were cultured on fibronectin-coated plates with EGM-2 medium (Lonza) with an extra 20 ng/mL of vascular endothelial growth factor (VEGF) (R&D Systems). For adipogenic differentiation, the phase-bright cells were cultured in DMEM with 10% FBS, 100 U/mL penicillin G, 100 μg/mL streptomycin, 2 mmol/L glutamine, 1 μM dexamethasone, 10 μg/ml insulin, 0.5 mM isobulyl-1-methylxanthione, and 200 μM indomethacin for 2 weeks and medium was changed every 3 days. For osteogenic differentiation, the phase-bright cells were cultured in osteogenic differentiation medium (Cambrex, PT-3002) for 2–3 weeks and medium was changed every 3 days as previously described^6^.

### Histological analysis

To investigate if BM-derived cells can acquire CSCs properties, GFP+ BM-derived cells and CSCs differentiation in BMT heart were tracked by immunostaing. Anti-GFP antibody (Invitrogen), anti-α-sarcomeric actin (α-SA) antibody (Sigma), anti-Sca-1 antibody (Abcam, Cambridge, MA), and anti-c-Kit antibody (Millipore, Billerica, MA) were used. Alexa Fluor 488, Alexa Fluor 594 and Alexa Fluor 647-conjugated secondary antibodies (all from Invitrogen) were applied appropriately. DAPI was used for nuclear counterstaining. To track transdifferentation of BM-derived cells, immunohistochemical staining of GFP expression was carried out. Briefly, the anti-GFP antibody was labeled with biotin-conjugated goat anti-rabbit IgG (Santa Cruz Biotech, Santa Cruz, CA) and the GFP expressions were detected by the color reaction with AEC Substrate Kit (BD Pharmingen).

### Myocardial infarction and cell delivery

To investigate if the BM-derived CSCs can be used for ischemia heart disease, ligation of the mid left anterior descending (LAD) artery for 30 minutes, and then followed by reperfusion was performed. 5 × 10^5^ GFP+ CSCs from BMT mice were injected intramyocardially into the peri-infarct zone of wild type FVB mice at two different sites with a total volume of 20 μl in each animal. Control animals received PBS injection instead. The survival and differentiation of transplanted CSCs were tracked by BLI and immunostaining.

## Additional Information

**How to cite this article**: Liu, N. *et al*. Bone Marrow Is a Reservoir for Cardiac Resident Stem Cells. *Sci. Rep*. **6**, 28739; doi: 10.1038/srep28739 (2016).

## Supplementary Material

Supplementary Information
