# Supplementary material for: Bone Marrow Is a Reservoir for Cardiac Resident Stem Cells
Source: Sci Rep. 2016 Jun 27;6:28739. doi: 10.1038/srep28739 (PMC4921812; doi:10.1038/srep28739)
Supplement: Supplementary Information [file srep28739-s1.pdf]

Bone Marrow Is a Reservoir for Cardiac Resident Stem Cells

Na Liu, Xin Qi, Zhibo Han, Lu Liang, Deling Kong, Zhongchao Han, Zuoxiang He, Zongjin Li

Supplemental Methods

Mouse breeding and genotyping. The FVB-GFP/Fluc mice, expressing firefly luciferase (Fluc) and green fluorescent protein (GFP), were obtained by mating FVB-GFP mice with FVB-Fluc mice. The luciferase transgenic mouse line (FVB-Luc), expressing firefly luciferase (Fluc) under the control of the widely expressed β-actin promoter, was kindly provided by Xenogen Corporation (Alameda, CA). The presence of Fluc was determined by PCR. The forward primer is 5'-TGGATTCTAAAACGGATTACCAGGG-3', and reverse primer is 5'-CCAAAACAACAACGGCGGC-3'. PCR conditions are: 97°C 5:00 min, 94.5^{o}C 0:40 min, 58 ℃ 1:30 min, 72 ℃ 1:30min for 35 Cycles, 72 ℃ 10:00 min, hold at 4 ℃ until analysis. The GFP transgenic mouse line (FVB-GFP), expressing GFP under the control of the widely expressed β-actin promoter, was kindly provided by Professor Baojin Wu[1]. The presence of GFP was determined by PCR. The forward primer is 5’-TCATGGCCGACAAGCAGAAGAACG-3’, and reverse primer is 5’-CGGCGGCGGTCACGAACTC-3’. PCR conditions are: 94 ℃ 5min, 94 ℃ 30s, 65 ℃ 30s, 72 ℃ 30s， 30 Cycles， 72 ℃ 10 min， hold at 4 ℃ until analysis. Wild type FVB mice were purchased from the Laboratory Animal Center of the Academy of Military Medical Sciences (Beijing, China). Protocols were approved by the Nankai University Animal Care and Use Committee guidelines, which conform to the Guide for the Care and Use of Laboratory Animals published by the US National Institutes of Health (8th edition, 2011).

Isolation and transplantation of bone marrow cells. Mice were anesthetized with inhaled isoflurane (2-3%). Eight to 10-week-old female FVB mice were irradiated with 12.0 Gy of γ-irradiation in 2 divided doses, 2 hours apart, on the day of surgery (n=70). Bone marrow cells were isolated from the femur and tibia of 8 to 10-week-old female transgenic FVB-GFP/Fluc mice (n=10), expressing the β-actin-GFP and firefly luciferase genes, by flushing with Iscove's minimal essential medium (IMEM). Red blood cells were lysised by BD™ Biosciences Lysing Buffer (BD Pharmingen) according to manufacturer's instructions. For bone marrow transplantation (BMT), wild-type irradiated FVB mice were injected with 0.2 ml PBS with or without 2.0×10^5 BM mononuclear cells via tail veins at 2 hours after irradiation. Mice were kept in a specific pathogen free facility and drinking water containing enrofloxacin (0.15 mg/ml) and amoxicillin (1 mg/ml) were given for 4 weeks to prevent infection. Chimerism of peripheral blood leukocytes was determined in transplanted mice by flow cytometry as described [2].

Transplantation of BM-derived CSCs into ischemia myocardium. All surgical procedures were performed on 8-10 week old female FVB mice (Laboratory Animal Center of the Academy of Military Medical Science, Beijing, China) by a single experienced micro-surgeon. Protocols were approved by the Nankai University Animal Care and Use Committee guidelines, which conform to the Guide for the Care and Use of Laboratory Animals published by the US National Institutes of Health (8th edition, 2011). Following induction with inhaled isoflurane (2-3%), mice were intubated and ventilated and anesthesia was maintained with inhaled isoflurane (1-2.5%). The ischemia/perfusion (I/R) surgery was done as previously reported[3, 4]. Briefly, cut the skin along the left side of sternum, and detach the subcutaneous tissues along the inferior fringe of

pectoralis major muscle. Then cut the pectoralis muscle groups transversely to expose the thoracic cage. After that, enter the fourth intercostal space by blunt dissection and separate the third and fourth ribs using micro-dissecting retractors. The left anterior descending (LAD) artery is localized 1–2 mm below the junction of pulmonary infundibulum and the left atrial appendage. We ligated the middle of LAD using a 5-0-polypropylene suture from the left border of the pulmonary conus to the right border of the left atrial appendage for 30 min. For intramyocardial injection, 5×10^5 CSCs are injected into two sites near the peri-infarct zone at 20 μl of total volume after LAD ligation (n=15). In control group, we injected the same volume of PBS solution to the mice (n=7). We monitored the mice until they are fully conscious, and then return them to their cages. During the operation, the heart rate, respiratory rate, and body temperature of the mice were carefully monitored. After surgery, buprenorphine (0.05/mg/kg, s.c.) was applied every 12 hours for 3 days for pain relief. At end of experiment, mice were sacrificed by exposure to 100% CO_2 followed by cervical dislocation, and heart samples were harvested for corresponding analyses.

Histological analysis. Hearts from study and control groups were embedded into OCT compound (Miles Scientific). Frozen sections (5 μm thick) were processed for immunostaining. To investigate if bone marrow-derived cells can acquire CSCs properties, GFP^+ BM-derived cells and CSCs differentiation in BMT heart were tracked by immunostaining. Rabbit anti-GFP antibody (Invitrogen), mouse anti-α-sarcomeric actin (α-SA) antibody (Sigma), rat anti-Sca-1 antibody (Abcam, Cambridge, MA), and rat anti-c-Kit antibody (Millipore, Billerica, MA) were used. Briefly, slices were incubated with first antibody for 1 h at room temperature and incubated

with goat anti-rabbit Alexa Fluor 488, goat anti-rat Alexa Fluor 594, or donkey anti-mouse Alexa Fluor 647 (all from Invitrogen) appropriately. DAPI was used for nuclear counterstaining.

To track transdifferentation of BM-derived cells in heart, immunohistochemical staining of GFP expression was also carried out at month 12 after BMT. Briefly, the anti-GFP antibody was labeled with biotin-conjugated goat anti-rabbit IgG (Santa Cruz Biotech, Santa Cruz, CA) and the GFP expressions were detected by the color reaction with AEC (3-Amino-9-Ethylcarbazole) Substrate Kit (BD Pharmingen). To detect endothelial differentiation of BM-derived cells, CD31 staining was carried out at month 12 after BMT. Rabbit anti-GFP antibody (Invitrogen) and rat anti-mouse CD31 (BD Pharmingen) were used. And Alexa Fluor 488 and 594-conjugated secondary antibody were used. To track transdifferentation of BM-derived cells in brain, skeletal muscle, intestine, and kidney, immunohistochemical staining of GFP expression was carried out at month 12 after BMT and nucleus were counter stained with DAPI.

To track cardiomyocytes differentiation of BM-derived CSCs in hearts at week 2 after cell transplantation (n=7), rabbit anti-GFP antibody (Invitrogen) and mouse anti-α-SA antibody (Sigma) were used. Alexa Fluor 488- and Fluor 594-conjugated secondary antibodies were applied appropriately. DAPI was used for nuclear counterstaining.

Supplemental References

1. Wu P, Yu L, Yin H, Yang W, Gu M, Liu G, WL. Z, Li G, Cao Y, Wu B. Breeding five kinds of congenic mice with the green fluorescent protein gene. *J Hangzhou Normal University (Natural Sciences Edition)*. 2013;12:8-13
2. Feng G, Mao D, Che Y, Su W, Wang Y, Xu Y, Fan Y, Zhao H, Kong D, Xu Y, Li Z. The phenotypic fate of bone marrow-derived stem cells in acute kidney injury. *Cell Physiol Biochem*. 2013;32:1517-1527
3. Su W, Leng L, Han Z, He Z, Li Z. Bioluminescence imaging of human embryonic stem cell-derived endothelial cells for treatment of myocardial infarction. In: Turksen K, ed. *Imaging and tracking stem cells*. Humana Press; 2013:1-13.
4. Li Z, Wilson KD, Smith B, Kraft DL, Jia F, Huang M, Xie X, Robbins RC, Gambhir SS, Weissman IL, Wu JC. Functional and transcriptional characterization of human embryonic stem cell-derived endothelial cells for treatment of myocardial infarction. *PLoS ONE*. 2009;4:e8443

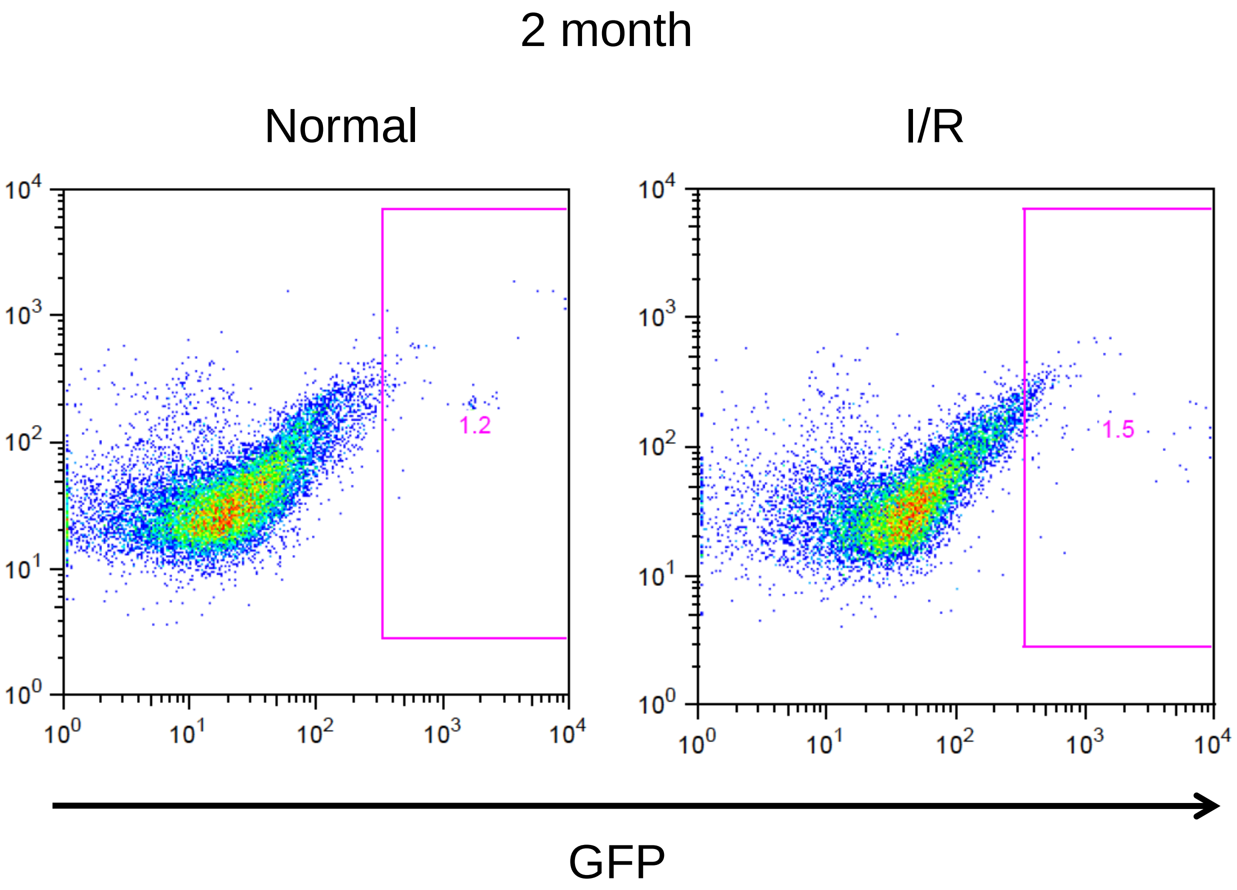

Figure S1. FACS analysis of GFP positive cells in heart after BMT with or without I/R injury at month 2. I/R could increase GFP^+ cells engraftment but not significant. Normal (n=4), IR (n=4).

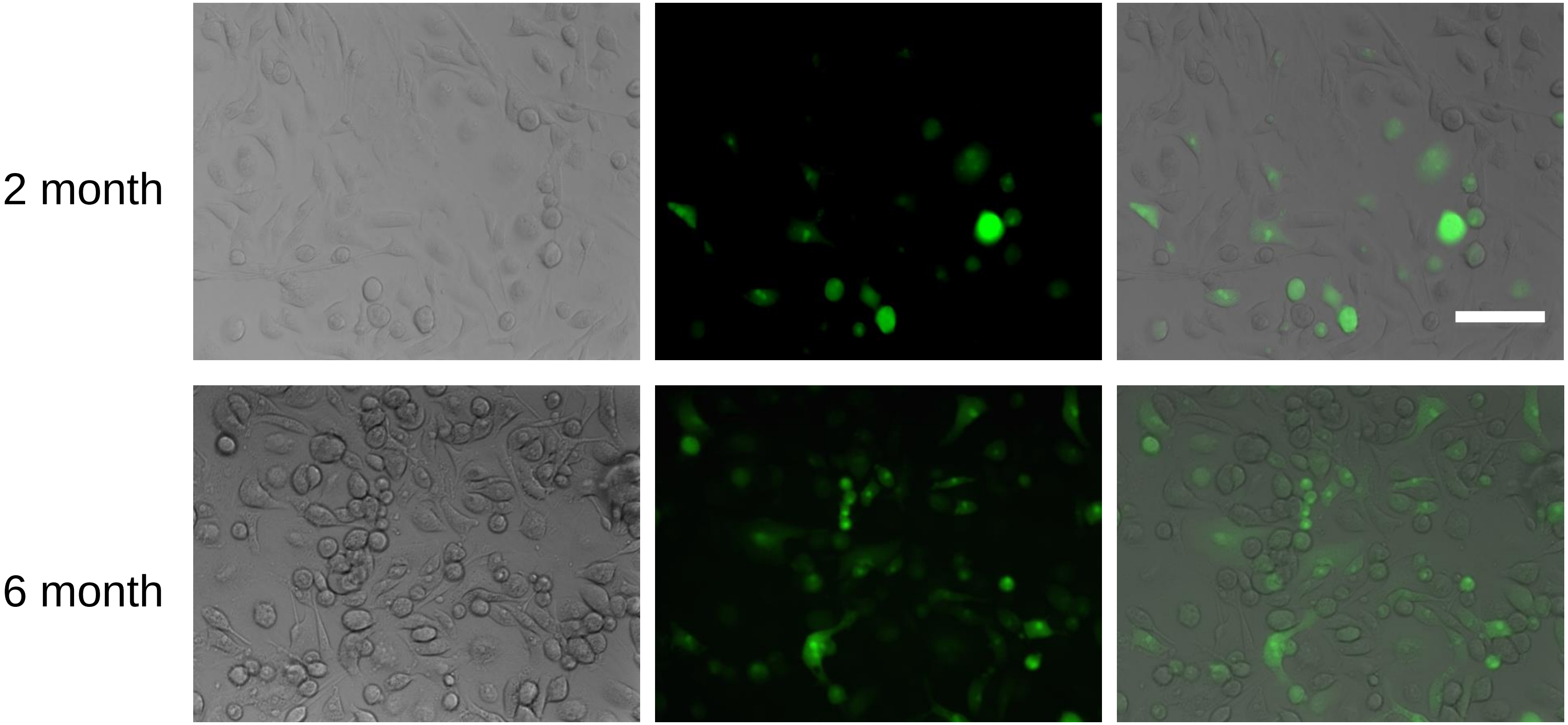

Figure S2. Subculture of phase-bright cells. The results revealed that GFP$^+$ cells were increased at month 6 compared to month 2 after BMT. Scale bar=50μm. Totally 10 hearts were harvested at month 6, and 4 hearts at month 2 for CSCs culture.

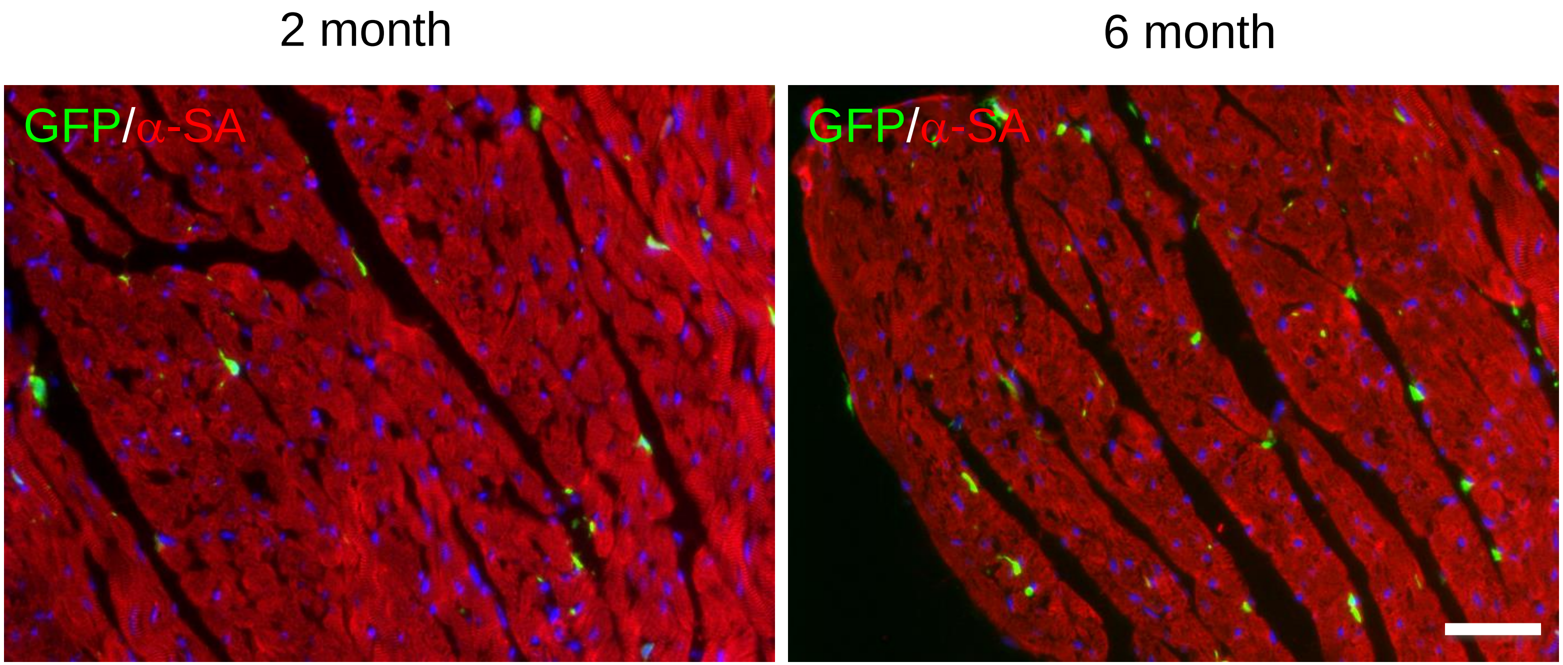

Figure S3. Engraftment of BM stem cells in heart. The results revealed that GFP+ cells were increased at month 6 compared to month 2 after BMT. Scale bar=50μm. Totally 10 hearts were harvested at month 6, and 4 hearts at month 2.

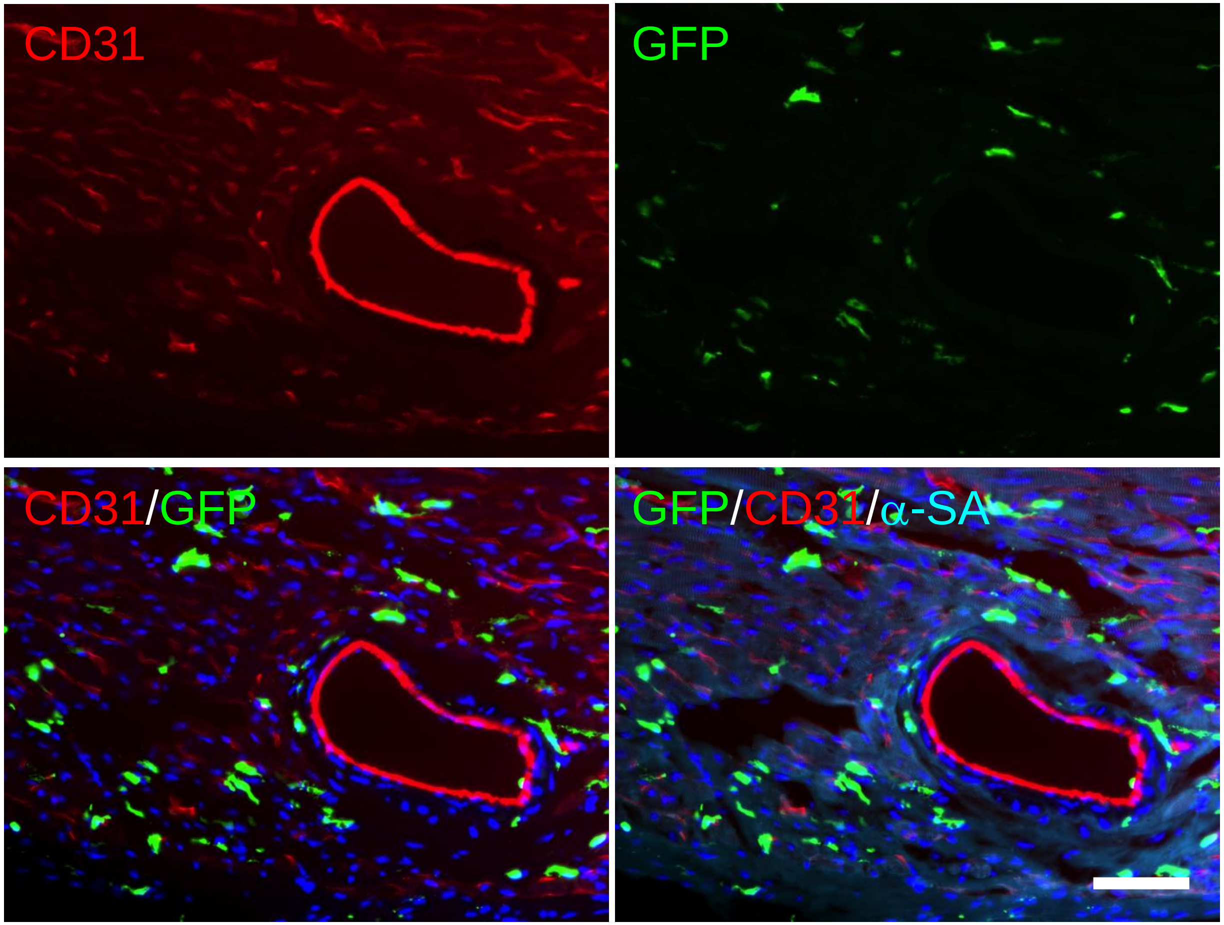

Figure S4. Endothelial differentiation of BM-derived cells. One year after BMT, BM-derived cells could differentiate into endothelial cells as confirmed by CD31 (red) and GFP (green) double staining. Cardiomyocytes were stained with α-sarcomeric actin (α-SA) (cyan) and nuclei were counter stained with DAPI (blue). Scale bar=50μm. Totally 10 hearts were harvested at month 12.

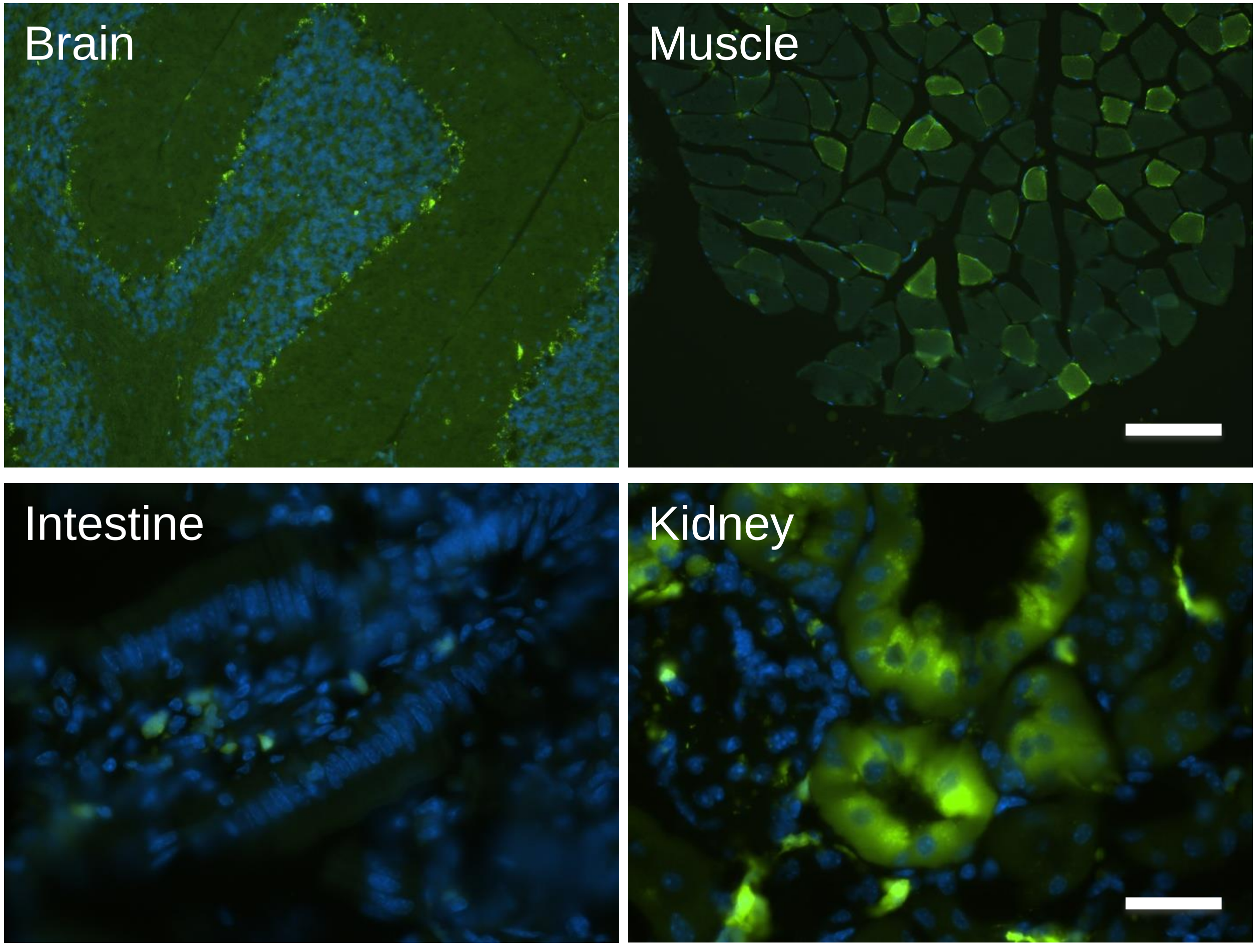

Figure S5. Transdifferentiation of BM-derived cells into other lineages. BM-derived cells could also transdifferentiate into Purkinje cells in cerebellum, skeletal muscles, villus cells in intestine, and renal tubule as confirmed by GFP stainings. Up panel, scale bar=200μm; Lower panel, scale bar=10μm. Totally 5 organs were harvested at month 12.
